# Does Bone Resorption Stimulate Periosteal Expansion? A Cross‐Sectional Analysis of β‐C‐telopeptides of Type I Collagen (CTX), Genetic Markers of the RANKL Pathway, and Periosteal Circumference as Measured by pQCT

**DOI:** 10.1002/jbmr.2093

**Published:** 2014-03-19

**Authors:** John P Kemp, Adrian Sayers, Lavinia Paternoster, David M Evans, Kevin Deere, Beate St Pourcain, Nicholas J Timpson, Susan M Ring, Mattias Lorentzon, Terho Lehtimäki, Joel Eriksson, Mika Kähönen, Olli Raitakari, Marika Laaksonen, Harri Sievänen, Jorma Viikari, Leo‐Pekka Lyytikäinen, George Davey Smith, William D Fraser, Liesbeth Vandenput, Claes Ohlsson, Jon H Tobias

**Affiliations:** ^1^ MRC Centre for Causal Analyses in Translational EpidemiologyUniversity of BristolBristolUK; ^2^ School of Social and Community MedicineUniversity of BristolBristolUK; ^3^ Center for Bone and Arthritis ResearchInstitute of Medicine, Sahlgrenska Academy, University of GothenburgGothenburgSweden; ^4^ Geriatric Medicine, Department of Internal Medicine and Clinical NutritionUniversity of GothenburgGothenburgSweden; ^5^ Department of Clinical Chemistry, Fimlab Laboratoriesand University of Tampere School of MedicineTampereFinland; ^6^ Department of Clinical PhysiologyUniversity of Tampere School of Medicine and Tampere University HospitalTampereFinland; ^7^ Research Centre of Applied and Preventive Cardiovascular MedicineUniversity of Turkuand the Department of Clinical Physiology and Nuclear MedicineTurku University HospitalTurkuFinland; ^8^ Department of Food and Environmental SciencesUniversity of HelsinkiHelsinkiFinland; ^9^ The UKK Institute for Health Promotion ResearchTampereFinland; ^10^ Department of MedicineUniversity of Turku and Turku University HospitalTurkuFinland; ^11^ Norwich Medical SchoolUniversity of East AngliaEast AngliaUK; ^12^ School of Clinical SciencesUniversity of BristolBristolUK

**Keywords:** CTX, BONE RESORPTION, PERIOSTEAL EXPANSION, pQCT

## Abstract

We hypothesized that bone resorption acts to increase bone strength through stimulation of periosteal expansion. Hence, we examined whether bone resorption, as reflected by serum *β*‐C‐telopeptides of type I collagen (CTX), is positively associated with periosteal circumference (PC), in contrast to inverse associations with parameters related to bone remodeling such as cortical bone mineral density (BMD_C_). CTX and mid‐tibial peripheral quantitative computed tomography (pQCT) scans were available in 1130 adolescents (mean age 15.5 years) from the Avon Longitudinal Study of Parents and Children (ALSPAC). Analyses were adjusted for age, gender, time of sampling, tanner stage, lean mass, fat mass, and height. CTX was positively related to PC (*β *= 0.19 [0.13, 0.24]) (coefficient = SD change per SD increase in CTX, 95% confidence interval)] but inversely associated with BMD_C_ (*β *= –0.46 [–0.52,–0.40]) and cortical thickness [*β *= –0.11 (–0.18, –0.03)]. CTX was positively related to bone strength as reflected by the strength‐strain index (SSI) (*β *= 0.09 [0.03, 0.14]). To examine the causal nature of this relationship, we then analyzed whether single‐nucleotide polymorphisms (SNPs) within key osteoclast regulatory genes, known to reduce areal/cortical BMD, conversely increase PC. Fifteen such genetic variants within or proximal to genes encoding receptor activator of NF‐κB (RANK), RANK ligand (RANKL), and osteoprotegerin (OPG) were identified by literature search. Six of the 15 alleles that were inversely related to BMD were positively related to CTX (*p* < 0.05 cut‐off) (*n* = 2379). Subsequently, we performed a meta‐analysis of associations between these SNPs and PC in ALSPAC (*n* = 3382), Gothenburg Osteoporosis and Obesity Determinants (GOOD) (*n* = 938), and the Young Finns Study (YFS) (*n* = 1558). Five of the 15 alleles that were inversely related to BMD were positively related to PC (*p* < 0.05 cut‐off). We conclude that despite having lower BMD, individuals with a genetic predisposition to higher bone resorption have greater bone size, suggesting that higher bone resorption is permissive for greater periosteal expansion. © 2014 The Authors. *Journal of Bone and Mineral Research* published by Wiley Periodicals, Inc. on behalf of the American Society for Bone and Mineral Research. This is an open access article under the terms of the Creative Commons Attribution License, which permits use, distribution and reproduction in any medium, provided the original work is properly cited.

## Introduction

Bone size and geometry make a major contribution to fracture risk, reflecting the fact that bending strength of bone is critically dependent on its diameter.^1^ Consistent with this view, we previously found that bone size relative to body size is a stronger protective factor against fractures in children compared with bone mineral density (BMD) measurements alone.^2^ Increases in bone diameter, accomplished through periosteal expansion, largely occur in childhood as part of the process of bone modeling. Gender differences in periosteal expansion (for example, in expansion of hip circumference during puberty^3^) may help to explain the higher prevalence of hip fractures in women compared with men in later life. Periosteal expansion is also thought to continue after longitudinal growth has ceased, although this subsequently declines in later life, limiting its ability to compensate for the higher resorption and endocortical expansion that characterizes bone loss in the elderly.^4^ This process may potentially protect from bone loss during aging. However, in spite of the importance of periosteal expansion to bone strength and fracture risk, this process has largely been ignored as a possible drug target, and we remain ignorant of many of the factors that influence it.^5^

One factor that may play a largely unrecognized role in periosteal expansion is bone resorption. As well as a role in bone remodeling, resorption is involved in growth and modeling, as evidenced by observations that periods of rapid growth such as puberty are associated with marked increases in resorption as well as formation markers.^6^ Remodeling is thought to be initiated by bone resorption, with bone formation occurring at sites of previous resorption.^7^ In contrast, increased resorption during growth is generally held to be secondary to increased bone formation. For example, resorption of the primary and secondary spongiosa occurs during endochondral bone formation at the growth plate, and resorption of cortical bone is required for adaptation of cortical bone shape and geometry to mechanical loading. However, it is not inconceivable that bone resorption plays a primary role in modeling as well as remodeling, such that periosteal expansion occurs secondary to increased resorption. For example, osteoclastic bone resorption also takes place at the periosteum.^4^ Moreover, it has been proposed that periosteal expansion represents part of an overall response intended to retain bone strength in the face of endosteal expansion.^4^

In the present study, we aimed to examine the contribution of bone resorption to periosteal expansion. We performed a cross‐sectional analysis of associations between *β*‐C‐telopeptides of type I collagen (CTX) and measures obtained from mid‐tibial peripheral quantitative computed tomography (pQCT) scans in a large group of adolescents from the Avon Longitudinal Study of Parents and Children (ALSPAC). Because bone modeling contributes a higher proportion to overall CTX levels in children compared with adults, this age group is ideally suited to analyzing relationships between resorption markers and phenotypes related to bone modeling. We aimed to determine whether CTX is positively associated with bone size as reflected by periosteal circumference (PC), in contrast to inverse associations with phenotypes such as cortical bone mineral density (BMD_C_) related to bone remodeling. Furthermore, we aimed to establish whether any positive association between CTX and PC reflects a causal pathway between bone resorption and periosteal expansion, by analyzing whether single‐nucleotide polymorphisms (SNPs) related to receptor activator of NF‐κB (RANK), RANK ligand (RANKL), and/or osteoprotegerin (OPG) presumed to increase bone resorption are also associated with greater PC.

## Materials and Methods

ALSPAC is a geographically based UK cohort that recruited pregnant women residing in Avon (southwest England) with an expected date of delivery between April 1, 1991, and December 31, 1992. A total of 15,247 pregnancies were enrolled with 14,775 children born (see www.alspac.bris.ac.uk for more information).^8^ Of these births, 14,701 children were alive at 12 months. The present study is based on research clinics to which the whole cohort was invited, held when participants were mean ages of 15.5 years. Ethical approval was obtained from the ALSPAC Law and Ethics committee, and the Local Research Ethics Committees. Parental consent and child's assent was obtained for all measurements made.

### Tibial pQCT

BMD_C_ and cortical bone mineral content (BMC_C_) of the mid (50% from the distal endplate) right tibia were obtained using a Stratec XCT2000L (Stratec, Pforzheim, Germany) during the age 15.5‐year research clinic to which all ALSPAC participants were invited as part of a study investigating the effects of physical activity on cortical bone as previously published.^10^ PC, endosteal circumference (EC), and cortical thickness (CT) were derived using a circular ring model. Cortical bone was defined using a threshold above 650 mg/cm^3^,^10^ and BMD_C_ subsequently derived. Strength strain index (SSI) was calculated according to the formula published by Hasegawa and colleagues.^11^

### Other variables

Height was measured using a Harpenden stadiometer (Holtain Ltd., Crymych, UK), and weight was measured to the nearest 50 g using Tanita weighing scales (Tanita UK Ltd., Uxbridge, UK). Data on lean mass and fat mass were obtained from total body dual‐energy X‐ray absorptiometry (DXA) scans performed at the age 15.5‐year clinic, using a Lunar Prodigy scanner (Lunar Radiation Corp., Madison, WI, USA) with pediatric scanning software (GE Healthcare Bio‐Sciences Corp., Piscataway, NJ, USA). Information on skeletal maturity was based on results of Tanner stage questionnaire at age 13.5 years (pubic hair domain), as previously found to be related to hip development as assessed by DXA.^3^ Electrochemiluminescence immunoassays (ECLIA) (Roche Diagnostics, Lewes, UK) were used to measure plasma concentrations of CTX on fasting samples collected at the age 15.5‐year clinic visit (detection limit 0.01 ng/mL), plasma being separated and frozen within 4 hours at –80°C. Inter‐ and intra‐assay coefficients of variation (CVs) were <6.0% across the working range.

### Genetic studies of periosteal circumference

We recently reported results for a genome‐wide meta‐analysis study for pQCT‐derived BMD_C_ involving three discovery cohorts, namely ALSPAC (*n* = 3382), The Gothenburg Osteoporosis and Obesity Determinants (GOOD) (*n* = 938), and Young Finns (YFS) (*n* = 1558).^12^ As part of the same study, a genome‐wide association study (GWAS) was also performed for PC, including age, gender, height, and weight (ln) as covariates, using additive linear regression in MACH2QTL for ALSPAC, ProbABEL^13^ for YFS, and MACH2QTL on GRIMP^14^ for the GOOD analyses. A meta‐analysis of the results from the three cohorts was then performed using the inverse variance method in METAL.^15^ Standardized betas and standard errors from each study were combined using a fixed effect model, which weights the studies using the inverse variance and applying genomic control to individual studies and the combined results. No associations were observed reaching genome‐wide significance (*p* < 5 × 10^−8^) (data not shown). Here, we compiled a list of SNPs related to RANK, RANKL, and OPG previously found to be associated with lumbar and/or hip areal BMD at a genome‐wide significance level, and looked up their association with PC, CT, and BMD_C_ in these three cohorts individually and after meta‐analysis (a RANKL SNP previously identified as being associated with BMD_C_ was also included^16^). We also looked up associations of these SNPs with CTX results in ALSPAC, based on the same model (with additional adjustment for time drawn). A description of GOOD and YFS participants included in this study and how pQCT and genetic data were collected are provided in the Supplemental Materials. {Suppl materials}

### Statistical analysis

Descriptive statistics are presented as means, standard deviations, and interquartile cut‐points. EC was adjusted for PC (EC_PC_) to derive a measure of relative cortical thickness. Ordinary least squares (OLS) linear regression was used to investigate: i) the relationship between known factors that influence the variation in serum concentrations of CTX; ii) the associations between CTX and pQCT variables after adjustment for average age (calculated from the age at scan and the age serum was drawn), time of clinic attendance (whether participants attended a morning or afternoon clinic, to take account of diurnal variation in CTX [samples largely clustered to within an hour of 8 a.m. or 12 p.m.]), gender, and Tanner stage (model 1); and iii) the effect of further adjustment for lean mass, fat mass, and height on model 1 (ie, model 2) (fat and lean mass were adjusted for in preference to weight, to account for the distinct relationship of these two compartments with cortical bone parameters).^10^ Because of the inverse association between BMD_C_ and PC, which we previously reported,^10^ results were also analyzed where BMD_C_ was adjusted for PC. Gender differences were explored by comparing *β* coefficients between separate analyses in males and females and by testing for gender interactions in analyses performed in males and females combined. A similar approach was used to examine the relationship between quartiles of CTX and the above‐mentioned pQCT measures, using the fully adjusted model (model 2). All analyses were conducted in STATA 11.1 MP (StataCorp, College Station, TX, USA).

## Results

### Description of participants

A total of 1130 participants (487 males, 643 females) were identified in ALSPAC with serum measurements of CTX and valid pQCT data at age 15.5 years. Height, weight, lean mass, and CTX were greater in males in contrast to fat mass, which was substantially greater in female participants (Table 1). BA_C_, BMC_C_, CT, PC, and SSI were greater in male participants, whereas BMD_C_ and EC_PC_ were higher in females.

**Table 1:** Characteristics of Participants Included in the Analysis of CTX and Tibial pQCT‐Derived Parameters as Mean, SD, Median, 25th (p25), and 75th (p75) Centiles

pQCT Variable	Male Mean	SD	p25	Median	p75	Female Mean	SD	p25	Median	p75
Age (years)	15.43	0.24	15.29	15.38	15.51	15.45	0.25	15.30	15.41	15.55
Height (cm)	173.91	6.97	169.30	174.40	178.70	164.62	5.82	161.10	164.40	168.30
F‐mass (kg)	10.04	6.78	5.72	8.14	11.95	17.65	6.58	13.04	16.44	21.46
L‐mass (kg)	49.54	6.19	45.53	49.46	53.82	37.01	3.66	34.68	36.75	39.46
Weight (kg)	62.39	10.00	56.20	61.50	67.50	57.70	8.73	51.90	56.60	62.80
CTX (ng/mL)	1.51	0.52	1.14	1.43	1.84	0.72	0.25	0.54	0.68	0.85
BAc (cm^2^)	328.75	42.74	298.75	329.54	356.72	275.23	34.90	252.93	273.98	297.15
BMCc (mg)	353.53	48.86	319.97	352.04	384.76	309.78	39.53	283.68	307.32	334.96
BMDc (mg/cm^3^)	1074.69	33.67	1055.56	1077.66	1097.27	1125.61	22.11	1111.37	1126.92	1140.16
BMDc‐adj‐PC (mg/cm^3^)	1084.51	35.75	1063.39	1088.20	1108.73	1116.55	22.74	1103.08	1117.68	1133.01
CT (mm)	5.62	0.62	5.21	5.63	6.02	5.20	0.54	4.82	5.21	5.56
EC (mm)	40.96	5.48	37.26	40.73	44.10	36.71	5.03	33.49	36.15	39.47
EC‐adj‐PC (mm)	38.44	3.81	36.02	38.37	40.65	38.91	3.32	36.76	38.78	40.98
PC (mm)	76.25	5.04	73.12	76.03	79.52	69.35	4.69	66.20	68.96	72.37
SSI (mm^3^)	1159.41	219.24	1005.68	1149.89	1301.66	918.48	172.95	801.30	895.20	1031.73

Age = mean age between age at pQCT scan and age when the blood sample was taken; CTX = β‐C‐telopeptides of type I collagen; F‐mass = total body fat mass; L‐mass = total body lean mass; BA_c_ = cortical bone area; BMC_C _= cortical bone mineral content; BMD_C_ = cortical bone mineral density; PC = periosteal circumference; CT = cortical thickness; EC = endosteal circumference; BMD_C_‐adj‐PC = cortical bone mineral content adjusted for periosteal circumference; EC‐adj‐PC = endosteal circumference adjusted for periosteal circumference; SSI = strength strain index.

Breakdown of participants according to Tanner stage and gender (male/female) at age 13.5 years: stage 1 (*n* = 49/30); stage 2 (*n* = 126/74); stage 3 (*n* = 135/145); stage 4 (*n* = 147/260); and stage 5 (*n* = 30/134).

### Associations between CTX and confounders

Age, gender, time of sampling, and Tanner stage all showed strong inverse associations with CTX (Supplemental Table S1, model 1). When height and weight were added, positive and inverse associations with CTX were observed, respectively. Weight was subsequently replaced by fat and lean mass, both of which showed independent inverse associations with CTX, although overall model fit was unchanged (Akaike information criterion = 2412, 2368, and 2369 for model 1, model 1 plus height and weight, and model 2, respectively).

### CTX versus tibial pQCT variables

**Figure 1:**
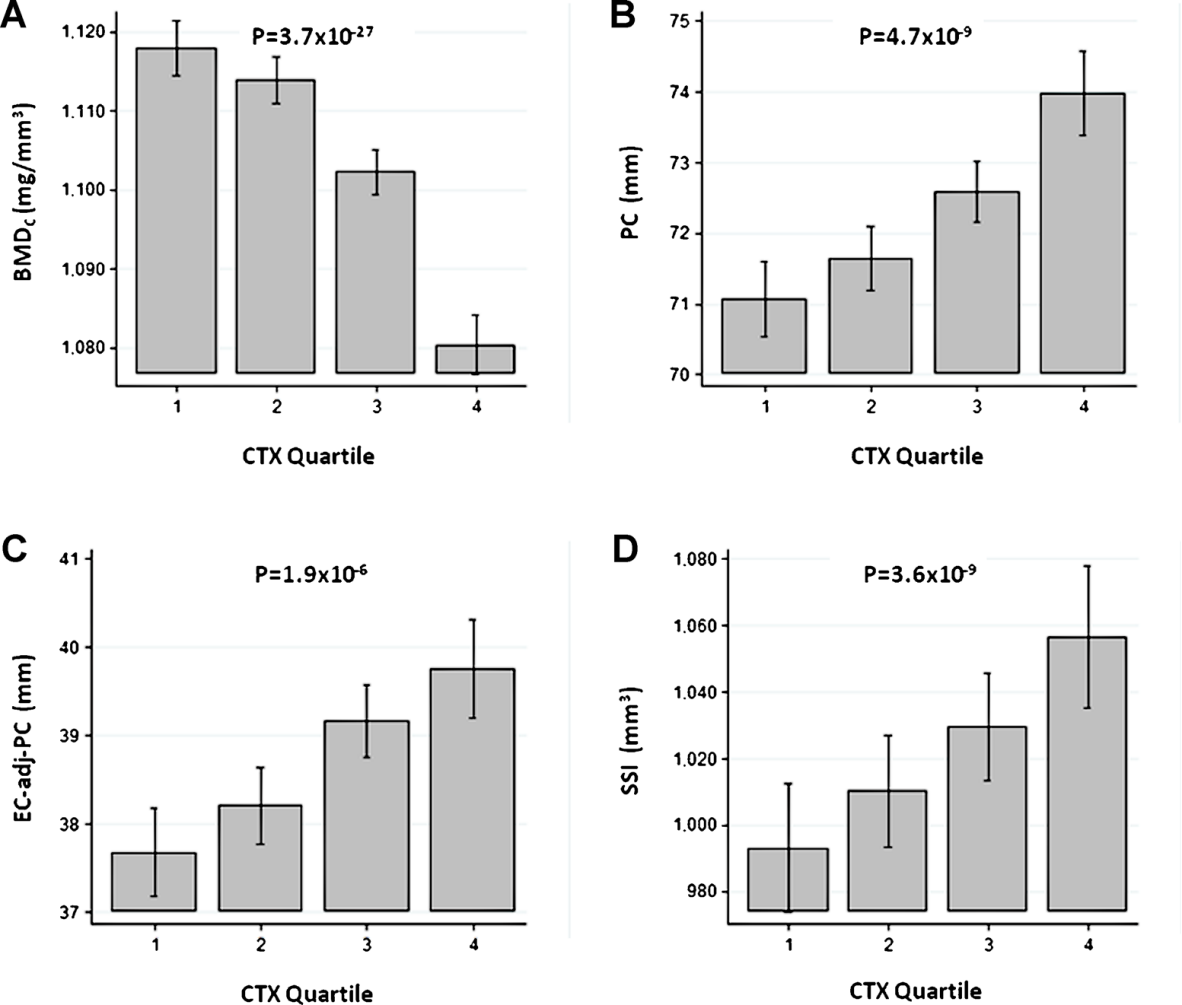
Relationships between quartiles of CTX and cortical BMD (*A*), periosteal circumference (*B*), endosteal circumference adjusted for periosteal circumference (*C*), and strength strain index (*D*). Data show mean > ± SD of each trait for each quartile of CTX at age 15 years, adjusted for average age, gender, Tanner stage, timing of sample collection, lean mass, fat mass, and height in 1130 individuals (boys = 487, girls = 643). *p* = strength of evidence against the null hypothesis of no association between the outcome and exposure variable.

In our minimally adjusted model (ie, model 1), CTX was unrelated to overall bone size as reflected by PC, but a strong positive association was observed after adjustment for body composition (ie, model 2 [Table 2]). Conversely, CTX appeared to be related to cortical thinning and increased cortical remodeling, as reflected by inverse associations with CT and BMD_C_, and a positive association with EC_PC_. CTX showed similar relationships with BMD_C_ and EC_PC_ in both models, whereas the association with CT showed partial attenuation after adjustment for body composition, as reflected by a 50% decrease in beta coefficients in model 2 versus model 1. Despite opposite relationships with PC and CT, there was a net positive association between CTX and BA_C_ and SSI (model 2). We also examined the relationship between CTX and pQCT measures by analyzing the latter variables, adjusted according to model 2, according to CTX quartile. Successive quartiles of CTX were associated with a linear increase in both PC and SSI (Fig. 1). Conversely, there was a progressive decline in BMD_C_, in which change was most marked for the top quartile. Increasing quartiles of CTX were also associated with a linear increase in EC_PC_. Throughout, similar results were obtained in males and females, as indicated by *p* > 0.1 for gender interaction.

**Table 2:** Regression Analyses of CTX versus pQCT Variables in 1130 Participants Aged 15.5 Years (487 Males, 643 Females)

Outcome	Model 1					Model 2				
	β	SE	L_CI	U_CI	*p Value*	β	SE	L_CI	U_CI	*p Value*
BAc	–0.12	0.036	–0.19	–0.05	**1.08 × 10**^**−3**^	0.06	0.026	0.01	0.12	**1.50 × 10**^**−2**^
BMC_C_	–0.22	0.038	–0.30	–0.15	**<0.001**	–0.03	0.028	–0.09	0.02	2.48 × 10^−1^
BMD_C_	–0.45	0.028	–0.51	–0.39	**<0.001**	–0.46	0.029	–0.52	–0.40	**<0.001**
BMD_C_‐adj‐PC	–0.49	0.032	–0.56	–0.43	**<0.001**	–0.42	0.031	–0.48	–0.36	**<0.001**
CT	–0.24	0.040	–0.32	−0.16	**<0.001**	–0.11	0.037	–0.18	–0.03	**4.27 × 10**^**−3**^
EC	0.20	0.045	0.12	0.29	**<0.001**	0.29	0.043	0.20	0.37	**<0.001**
EC‐adj‐PC	0.28	0.045	0.20	0.37	**<0.001**	0.22	0.045	0.13	0.31	**<0.001**
PC	0.02	0.038	–0.05	0.09	6.07 ×** **10^−1^	0.19	0.029	0.13	0.24	**<0.001**
SSI	–0.10	0.038	–0.18	–0.03	**8.51 × 10**^**−3**^	0.09	0.027	0.03	0.14	**1.32 × 10**^**−3**^

Model 1 = adjustment for age, gender, whether the individual attended the clinic in the morning or afternoon, and Tanner stage. Model 2 = Model 1 in addition to lean mass, fat mass, and height.

SE = standard error; *β* = SD change in outcome per SD increase in CTX; L_CI = lower 95% confidence estimate of *β*; U_CI = upper 95% confidence estimate of *β*; *p* = strength of evidence against the null hypothesis of no association between the outcome and exposure variable; BA_C_ = cortical bone area; BMC_C_ = cortical bone mineral content; BMD_C_ = cortical bone mineral density; PC = periosteal circumference; CT = cortical thickness; EC = endosteal circumference; BMD_C_‐adj‐PC = cortical bone mineral content adjusted for periosteal circumference; EC‐adj‐PC = endosteal circumference adjusted for periosteal circumference; SSI = strength strain index.

### Exploration of causal pathways

#### Selection of genetic instruments for bone resorption

**Figure 2:**
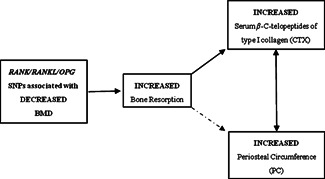
Proposed causal pathway between bone resorption and periosteal circumference. Solid arrows depict relationships assumed a priori. Two‐headed arrow represents the observed association between CTX and periosteal circumference. Dashed arrow represents the hypothetical causal pathway between bone resorption and periosteal circumference to be explored.

We hypothesized that the positive association between CTX and PC reflects a causal pathway whereby increased bone resorption leads to an increase in periosteal expansion (Fig. 2). We explored this question using a Mendelian Randomization approach,^17^ based on genetic instruments that reflect constitutive determinants of bone resorption. To our knowledge, no genetic markers have been robustly associated with either CTX or any other resorption marker. Therefore, we adopted an alternative strategy using SNPs within and immediately adjacent to the genes for RANK, RANKL, and OPG, which have previously been reported to be associated with areal/cortical BMD in genome‐wide meta‐analysis, on the assumption that these associations are likely to be mediated by genetic effects on bone resorption. Seven OPG, four RANKL, and two RANK gene SNPs (including SNPs immediately adjacent to these genes) were identified as being associated with lumbar spine and/or femoral neck areal BMD from genome‐wide meta‐analyses^18^ (Table 3). One further independent OPG SNP (ie, rs7839059) and one further independent RANKL SNP (ie, rs1021188) were included on the basis of previous reported association with BMD_C_.^16^

**Table 3:** Evidence of Association of Previously Reported RANK (*TNFRSF11A*), RANKL (*TNFSF11*), and OPG (*TNFRSF11B*) Variants With CTX (ALSPAC Only, *n* = 2379)

							CTX			Lumbar spine BMD			Femoral neck BMD		
Locus	RSID	Pos	Gene	PMID	r^2^	EA	β*	SE	*p Value*	β*	SE	*p Value*	β*	SE	*p Value*
8q24.12	rs4355801	119993054	*TNFRSF11B*	19079262 & 18455228	0.90	A	0.02	0.030	4.81 × 10^−1^	–0.07	0.009	**8.09 × 10**^**−17**^	–0.06	0.008	**5.48 × 10**^**−12**^
	rs7839059	120045723	*TNFRSF11B*	23437003	*0.42*	A	0.07	0.031	**2.93 × 10**^**−2**^	–*0.08*	*0.009*	***1.45 *× *10***^***−16***^	–*0.07*	*0.009*	**4.73 × 10**^**−17**^
	rs2062375	120046973	*TNFRSF11B*	20548944	0.97	C	0.04	0.030	1.75 × 10^−1^	–0.08	0.009	**2.52 × 10**^**−20**^	–0.06	0.008	**3.35 × 10**^**−15**^
	**rs2062377**^a^	**120076601**	***TNFRSF11B***	**19801982 & 22504420**	**1.00**	**A**	–0.04	0.030	1.47 × 10^−1^	–0.08	0.009	**2.26 × 10**^**−20**^	–0.06	0.008	**2.50 × 10**^**−14**^
	rs6469792	120077552	*TNFRSF11B*	19079262	0.74	C	0.03	0.029	3.93 × 10^−1^	–0.08	0.009	**3.12 × 10**^**−20**^	–0.07	0.008	**2.38 × 10**^**−16**^
	rs11995824	120081881	*TNFRSF11B*	19801982	0.79	G	0.03	0.030	3.58 × 10^−1^	–0.08	0.009	**5.33 × 10**^**−20**^	–0.07	0.008	**1.02 × 10**^**−16**^
	rs6469804	120114010	*TNFRSF11B*	18445777 & 19079262	0.88	A	0.05	0.030	1.09 × 10^−1^	–0.08	0.009	**5.08 × 10**^**−18**^	–0.06	0.008	**1.94 × 10**^**−11**^
	rs6993813	120121419	*TNFRSF11B*	18445777 & 19079262	0.69	C	0.03	0.030	3.12 × 10^−1^	–0.08	0.009	**1.45 × 10**^**−18**^	–0.06	0.008	**6.41 × 10**^**−14**^
13q14.11	**rs9533090**^a^	**41849449**	***AKAP11***	**19801982 & 22504420**	**1.00**	**T**	0.09	0.029	**3.32 × 10**^**−3**^	–0.11	0.009	**1.02 × 10**^**−35**^	–0.05	0.008	**9.84 × 10**^**−11**^
	rs9594738	41850145	*TNFSF11*	18445777 & 19079262	1.00	T	0.09	0.029	**3.30 × 10**^**−3**^	–0.11	0.009	**6.46 × 10**^**−35**^	–0.05	0.008	**1.27 × 10**^**−10**^
	rs9533093	41859597	*TNFSF11*	19079262	0.23	T	0.02	0.034	5.78 × 10^−1^	–0.07	0.010	**1.67 × 10**^**−11**^	–0.03	0.010	**5.77 × 10**^**−4**^
	rs9594759	41930593	*TNFSF11*	18445777 & 19079262	0.68	T	0.04	0.029	1.50 × 10^−1^	–0.07	0.009	**4.66 × 10**^**−15**^	–0.03	0.008	**1.25 × 10**^**−3**^
	rs1021188^a^	42014133	*TNFSF11*	21124946 & 23437003	*0.00*	C	0.14	0.038	**4.12 × 10**^**−4**^	–*0.03*	*0.011*	***9.51 *× *10***^***−3***^	–*0.02*	*0.010*	***1.71 *× *10***^***−2***^
18q21.33	**rs884205**^a^	**58205837**	***TNFRSF11A***	**19801982 & 22504420**	**1.00**	**A**	0.11	0.034	**1.14 × 10**^**−3**^	–0.06	0.011	**4.85 × 10**^**−9**^	–0.04	0.010	**3.87 × 10**^**−5**^
	rs3018362	58233073	*TNFRSF11A*	19079262	0.68	A	0.07	0.031	**3.78 × 10**^**−2**^	–0.04	0.009	**7.15 × 10**^**−6**^	–0.03	0.009	**7.30 × 10**^**−5**^

Pos = position in the genome based on hg18; Gene = closest gene; PMID = accession number of the publication in Pubmed, which described the association with BMD; r^2^ = the pairwise LD estimate in CEU populations between the SNP in bold and all other SNPs in that locus; EA = effect allele; β* = effect size; SE = standard error of β*.

* Effect estimates expressed as adjusted SD per copy of the effect allele (EA). Note: rs9533090 is found upstream of *TNFSF11* but is closest to *AKAP11*. Data are also shown for lumbar spine and femoral neck BMD associations from the GEFOS publicly released data set (rs7839059 and rs1021188 were identified from a GWAS meta‐analysis based on BMD_C_ as opposed to areal BMD).

a Denotes the variants that were used to generate allele scores (ie, independent signals [rs1021188 was also included by virtue of genome‐wide significant association with BMDc]).

#### Genetic association studies in ALSPAC

We investigated whether associations between the 15 RANK, RANKL, and OPG SNPs and areal/cortical BMD, described in Table 3, are likely to be mediated by genetic effects acting to increase bone resorption. Six of these 15 alleles were related to CTX in the opposite direction to areal/cortical BMD (rs7839059 [OPG], rs9533090, rs9594738, rs1021188 [RANKL], rs884205, rs3018362 [RANK]), based on a *p* < 0.05 cut‐off for nominal significance (Table 3). Subsequently, we analyzed associations between RANK/RANKL/OPG SNPs and pQCT parameters. Five of the 15 RANK/RANKL/OPG SNPs were related to CT, the risk alleles being the same as those for areal/cortical BMD in all cases (Supplemental Table S2). Similarly, 9 of the 13 SNPs previously reported to be associated with areal BMD were also related to BMD_C_, with equivalent risk alleles for each trait (Supplemental Table S3).

We then explored a possible causal pathway between bone resorption and periosteal expansion by examining whether RANK/RANKL/OPG SNPs associated with areal/cortical BMD are also associated with PC. In particular, we wished to determine whether alleles that are inversely related to BMD (indicating greater bone resorption) are positively related to PC. Interestingly, three of these RANK/RANKL/OPG SNPs were associated with PC (*p* < 0.05), the direction of effect being opposite to that observed for areal/cortical BMD in all instances (Supplemental Table S4).

Similar findings were obtained based on allele scores generated without prior knowledge of the individual SNP tests. These scores were constructed using allele counts from top hits within the three loci as reported by Estrada and colleagues.^19^ The three SNPs selected are highlighted in bold (Table 3), along with rs1021188, which was also selected on the basis that this SNP represents a further independent genetic influence. An increase in CTX (*β *= 0.07 [0.05, 0.10] and *p *= 5.3 × 10^−8^), decrease in BMD_C_ (*β *= –0.09 [–0.11, –0.06] and *p *= 1.5 × 10^−11^), decrease in CT (*β *= –0.05 [–0.07, –0.02] and *p *= 0.002), and increase in PC (*β *= 0.03 [0.01, 0.05] and *p *= 0.01] were observed per unit increase in areal/cortical BMD risk allele score. After exclusion of rs2062377 from generation of allele scores, on the basis that this was unrelated to CTX, similar results were obtained: each unit increase in risk allele score was associated with an increase in CTX (*β *= 0.07 [0.05, 0.10] and *p *= 4.1 × 10^−8^], decrease in BMD_C_ (*β *= –0.07 [–0.10, –0.05] and *p *= 5.4 × 10^−9^), decrease in CT (*β *= –0.04 [–0.07, –0.01] and *p *= 0.01), and increase in PC (*β *= 0.02 [0.002, 0.04] and *p *= 0.03).

#### Replication studies in other cohorts

We looked up associations between the 15 RANK/RANKL/OPG SNPs related to areal/cortical BMD and pQCT traits from our GWAS involving two other cohorts, namely GOOD and YFS. In YFS, all 8 OPG SNPs showed an equivalent relationship with CT to that observed for areal/cortical BMD (Supplemental Table S2); 9 of the 13 SNPs associated with areal BMD showed equivalent relationships with BMD_C_ (Supplemental Table S3); 9 of the 15 areal/cortical BMD SNPs showed opposite associations with PC (Supplemental Table S4). Equivalent findings were observed in GOOD in respect of BMD_C_ associations, whereas there was less evidence for associations with CT and PC.

Finally, we performed a meta‐analysis of associations between the 15 RANK/RANKL/OPG SNPs and pQCT parameters across all three cohorts. Nine of these SNPs were associated with CT (Table 4). Of the 13 SNPs previously identified as being associated with areal BMD, 11 were also associated with BMD_C_. These associations were stronger for BMDc than for CT. The risk allele was equivalent when comparing areal BMD with BMD_C_/CT in all instances apart from rs9533093, which was unique in being associated with CT but not BMD_C_ and may have represented a false positive. Five of the 15 alleles related to areal/cortical BMD were associated with PC, the direction of effect being opposite to that for BMD in all cases (rs4355801, rs2062375 [OPG]; rs884205, rs3018362 [RANK], rs1021188 [RANKL]) (*p* < 0.05) (Table 4). Three further alleles showed evidence of a weak association with PC in the opposite direction to areal and cortical BMD (rs7839059, rs2062377, rs6469804 [OPG]) (*p* < 0.1).

**Table 4:** Evidence of Association of Previously Reported RANK (*TNFRSF11A*), RANKL (*TNFSF11*), and OPG (*TNFRSF11B*) Variants With Periosteal Circumference (PC), Cortical Thickness (CT), and Cortical Bone Mineral Density (BMD_C_), Obtained From Genome‐Wide Meta‐Analysis From ALSPAC (*n* = 3382), GOOD (*n* = 938), and Young Finns (*n* = 1558) (ie, total = 5878)

							PC			CT			BMDc		
Locus	RSID	Pos	Gene	PMID	r^2^	EA	β*	SE	*p Value*	β*	SE	*P*	β*	SE	*p Value*
8q24.12	rs4355801	119993054	*TNFRSF11B*	19079262 & 18455228	0.90	A	0.04	0.014	**3.96 × 10**^**−3**^	–0.01	0.017	5.14 × 10^−1^	–0.08	0.015	**8.75 × 10**^**−7**^
	rs7839059	120045723	*TNFRSF11B*	23437003	0.42	A	*0.03*	*0.014*	*6.70 × 10*^*−2*^	–0.04	0.018	**1.62 × 10**^**−2**^	–0.10	0.016	**4.14 × 10**^**−9**^
	rs2062375	120046973	*TNFRSF11B*	20548944	0.97	C	0.03	0.014	**2.14 × 10**^**−2**^	–0.04	0.017	**3.89 × 10**^**−2**^	–0.09	0.015	**4.81 × 10**^**−9**^
	**rs2062377**^a^	**120076601**	***TNFRSF11B***	**19801982 & 22504420**	**1.00**	**A**	0.02	0.014	8.91 × 10^−2^	–0.03	0.017	8.82 × 10^−2^	–0.08	0.016	**1.42 × 10**^**−7**^
	rs6469792	120077552	*TNFRSF11B*	19079262	0.74	C	0.02	0.014	1.75 × 10^−1^	–0.04	0.017	**2.30 × 10**^**−2**^	–0.07	0.015	**3.12 × 10**^**−6**^
	rs11995824	120081881	*TNFRSF11B*	19801982	0.79	G	0.02	0.014	1.36 × 10^−1^	–0.04	0.017	**1.05 × 10**^**−2**^	–0.08	0.016	**1.97 × 10**^**−7**^
	rs6469804	120114010	*TNFRSF11B*	18445777 & 19079262	0.88	A	0.02	0.014	7.15 × 10^−2^	–0.03	0.017	9.27 × 10^−2^	–0.08	0.016	**4.20 × 10**^**−7**^
	rs6993813	120121419	*TNFRSF11B*	18445777 & 19079262	0.69	C	0.02	0.014	1.04 × 10^−1^	–0.04	0.017	**1.60 × 10**^**−2**^	–0.08	0.016	**4.05 × 10**^**−7**^
13q14.11	**rs9533090**^a^	**41849449**	***AKAP11***	**19801982 & 22504420**	**1.00**	**T**	0.00	0.014	9.30 × 10^−1^	0.01	0.017	7.15 × 10^−1^	–0.04	0.016	**1.98 × 10**^**−2**^
	rs9594738	41850145	*TNFSF11*	18445777 & 19079262	1.00	T	0.00	0.014	9.06 × 10^−1^	0.01	0.017	7.32 × 10^−1^	–0.04	0.015	**1.54 × 10**^**−2**^
	rs9533093	41859597	*TNFSF11*	19079262	0.23	T	–0.01	0.016	6.00 × 10^−1^	0.05	0.020	**1.74 × 10**^**−2**^	–0.01	0.018	5.02 × 10^−1^
	rs9594759	41930593	*TNFSF11*	18445777 & 19079262	0.68	T	–0.01	0.014	5.52 × 10^−1^	0.00	0.017	8.93 × 10^−1^	0.00	0.015	9.53 × 10^−1^
															
	rs1021188^a^ †	42014133	*TNFSF11*	21124946 & 23437003	0.00	C	*0.05*	*0.018*	***3.93 *× *10***^***−3***^	–0.07	0.023	**2.86 × 10**^**−3**^	–0.15	0.021	**1.46 × 10**^**−12**^
18q21.33	**rs884205**^a^	**58205837**	***TNFRSF11A***	**19801982 & 22504420**	**1.00**	**A**	0.04	0.016	**5.60 × 10**^**−3**^	–0.06	0.019	**1.43 × 10**^**−3**^	–0.06	0.018	**6.02 × 10**^**−4**^
	rs3018362	58233073	*TNFRSF11A*	19079262	0.68	A	0.04	0.014	**1.30 × 10**^**−2**^	–0.05	0.018	**7.46 × 10**^**−3**^	–0.06	0.016	**1.84 × 10**^**−4**^

POS = position in the genome based on hg18; GENE = closest gene; PMID = accession number of the publication in Pubmed, which described the association with BMD; r^2^ = the pairwise LD estimate in CEU populations between the SNP in bold and all other SNPs in that locus; EA = effect allele; β* = effect size; SE = standard error of β*.

* Effect estimates expressed as adjusted SD per copy of the effect allele (EA). Note: rs9533090^a^ is found upstream of *TNFSF11* but is closest to *AKAP11*.

a Denotes the variants that were used to generate allele scores (ie, independent signals).

## Discussion

In a large cohort of adolescents, we found CTX to be positively related to periosteal expansion as reflected by PC but inversely related to BMD_C_ and CT. The associations between CTX and PC and BMD_C_ were particularly striking, such that a one‐SD increase in CTX was associated with 0.19 and −0.46 SD changes in these parameters, respectively. In spite of the finding that higher CTX was related to lower CT, the positive relationship between CTX and PC translated into a positive relationship with BA_C_. A positive relationship was also observed between CTX and predicted bone strength as estimated by SSI, reflecting the fact that the latter parameter is strongly influenced by bone size. Although SSI is also determined by BMD_C_, the inverse relationship between CTX and BMD_C_ did not appear to be sufficient to offset the positive relationship between CTX and SSI.

The positive association between CTX and bone area and PC was only evident after adjusting for body size, reflecting a separate inverse association between CTX and bone size acting via weight. The latter pathway reflects two distinct components. We have previously reported positive associations between fat and lean mass, and bone area and PC as measured by DXA and pQCT respectively.^10^ Furthermore, there appeared to be an inverse association between CTX and fat and lean mass, consistent with previous reports of an inverse association between fat mass and another turnover marker, osteocalcin.^24^ Although we are not aware of equivalent reports of associations of CTX with lean mass, possibly this relationship reflects the pathway between bone turnover and energy balance previously suggested by Karsenty and colleagues, thought to be mediated by osteocalcin.^25^

To explore the causal nature of the association between CTX and PC, we applied an instrumental variable approach, as previously used to examine the relationship between fat mass and bone mass.^27^ Although bone resorption was evaluated by measurement of plasma CTX, to our knowledge, no genetic markers for CTX are available. Therefore, we selected genetic instruments on the basis of i) known biological role in bone metabolism restricted to bone resorption, and ii) established association with areal/cortical BMD. RANKL/RANK/OPG was ideally suited for this purpose; this pathway plays a major role in regulating osteoclast differentiation, to which its biological effects are restricted as evidenced by extensive animal and clinical data;^28^ SNPs related to RANKL/RANK/OPG are not only robustly associated with areal BMD, but also this is one of the three key pathways found to explain genetic variability of this trait.^19^ Consistent with the suggestion that SNPs related to RANKL/RANK/OPG can be used as genetic instruments for bone resorption, the rs1021188 RANKL SNP has previously been related to RANKL expression^23^ and to cortical porosity as measured by high‐resolution peripheral quantitative computed tomography (HR‐pQCT).^13^ Moreover, in the present study, a large proportion of RANK/RANKL/OPG SNPs showed equivalent associations with CTX levels, BMD_C_, and CT to those seen for areal BMD. Even in the case of those RANK/RANKL/OPG SNPs that showed little evidence of association with CTX, we assume that their association with BMD is mediated by altered levels of bone resorption, given that the role of the RANK/RANKL/OPG system is restricted to regulation of osteoclast function.

Therefore, our observation that a substantial proportion of these SNPs are also related to PC, such that a risk allele for areal/cortical BMD is associated with greater PC, raises the possibility that a causal pathway exists between increased bone resorption and greater periosteal expansion. Areal BMD, which was used to identify 13 of our 15 genetic instruments, is positively associated with traits such as PC, which reflect bone size.^30^ Therefore, any phenotypic correlation between areal BMD and PC is unlikely to provide an alternative explanation for our findings because this would result in genetic markers of lower areal BMD having a lower PC, in contrast to a higher PC as reported here. On the other hand, we previously reported an inverse association between BMD_C_ and PC,^10^ which may help to explain why the two SNPs (rs1021188 and rs7839059) associated with lower BMD_C_ were associated with higher PC. This is made more likely by the fact that these two SNPs were originally identified in a meta‐analysis based on the same cohorts used to examine associations with PC. However, BMD_C_ is strongly influenced by cortical porosity, which in turn reflects bone remodeling, consistent with the strong inverse relationship we observed between BMD_C_ and CTX. Therefore, rather than providing a spurious association, the phenotypic correlation between BMD_C_ and PC is likely to be a direct consequence of our hypothesized causal pathway between bone resorption and periosteal expansion.

Taken together, our results suggest that although individuals with a constitutive predisposition to higher rates of bone resorption have a lower areal and/or cortical BMD, any adverse effect on bone strength and fracture risk may be at least partially compensated for by greater bone size. One possible mechanistic explanation for this relationship is that periosteal expansion occurs as a compensatory response to increased endosteal expansion as part of the mechanostat, thereby serving to maintain strains within cortical bone within a target range.^31^ However, if this explanation was responsible, one might have expected that CTX would have no net association with BA_C_ or SSI once compensatory changes in PC are taken into account, in contrast to the positive associations, which we observed with these parameters. Although the associations between CTX and bone size relative to body size that we report are limited to adolescents, our genetic study encompassed older individuals and raise the possibility that positive relationships between CTX and bone size persist into later life. Consistent with this suggestion, if anything, associations between RANK/RANKL/OPG SNPs and PC were strongest in YFS, which comprises adults aged 31 to 46 years.

Any tendency for higher bone resorption to be permissive for greater bone expansion may have implications for bone strength and fracture risk. For example, in a previous prospective study, we found that bone size relative to body size is an important protective factor for fracture risk in childhood.^2^ Higher rates of bone resorption in childhood may also protect against fracture risk in later life as a consequence of effects on bone size. For example, higher rates of bone resorption in boys compared with girls during puberty and adolescence, as noted here and previously,^32^ may contribute to the greater periosteal expansion of the hip that occurs at this time in boys compared with girls;^3^ this may in turn contribute to the lower fracture risk of males compared with females in later life. Similarly, reports that CTX is reduced in children and adolescents with type I diabetes^34^ suggest that reduced modeling contributes to the increased risk of hip fractures seen in this condition in later life.^35^

### Limitations

Our cross‐sectional study in ALSPAC was limited in that CTX is the only bone turnover marker measured in this cohort to date. Because formation and resorption markers are both produced during growth, modeling, and remodeling, equivalent results are likely to have been obtained based on bone formation markers that are also produced as part of these processes. Because CTX is a marker of type I collagen, which is not restricted to bone, it would also have been preferable to confirm our findings based on another resorption marker. A further limitation is that this study was based on a subset of the original ALSPAC cohort and is therefore likely to differ from a truly representative population sample in several ways. We ran further models adjusting for other confounders such as physical activity, based on contemporaneous accelerometer recordings, which did not materially affect the results (data available on request). However, we were unable to adjust for other potential confounders that were not measured, such as contemporaneous levels of 25‐hydroxyvitamin D3. In terms of limitations to our genetic analyses, although SNPs related to RANK/RANKL/OPG were used as genetic instruments for bone resorption, we are unable to exclude the possibility that these SNPs may have influenced BMD via other pathways. Few alternative genetic instruments for bone resorption exist to enable confirmation of our findings based on other pathways. Rs13336428, which is related to the *CLCN7* gene for the osteoclast chloride channel required for bone resorption, was reported to be associated with areal BMD,^19^ but we observed no association between this SNP and CTX in ALSPAC (data available on request).

Having investigated associations between CTX and pQCT measurements from the mid‐tibia in adolescents aged 15.5 years, bone resorption was found to be inversely related to traits reflecting lower rates of bone remodeling such as BMD_C_ but positively related to traits reflecting greater bone modeling such as PC. It is well established that bone resorption is the primary determinant of bone remodeling, but its relationship with bone modeling, which we went on to explore using a genetics approach, is less clear cut. Interestingly, genetic factors that predispose to greater bone resorption were also found to predispose to greater PC, raising the possibility that higher bone resorption is permissive for greater periosteal expansion. In light of these findings, further studies are justified to examine whether systemic markers of bone resorption might prove useful in monitoring adverse effects of pharmacotherapy and disease states on skeletal modeling, particularly during periods when this process is most active, such as during childhood and adolescence.

## Disclosures

All authors state that they have no conflicts of interest.

## Supplementary Material

Additional Supporting Information may be found in the online version of this article.

Supplementary Tables.
