# Supplementary material for: Does Bone Resorption Stimulate Periosteal Expansion? A Cross‐Sectional Analysis of β‐C‐telopeptides of Type I Collagen (CTX), Genetic Markers of the RANKL Pathway, and Periosteal Circumference as Measured by pQCT
Source: J Bone Miner Res. 2014 Mar 19;29(4):1015–24. doi: 10.1002/jbmr.2093 (PMC4138988; doi:10.1002/jbmr.2093)
Supplement: Supplementary file 1 — Supplementary Tables. [file jbmr-29-1015-s3.pdf]

Supplementary Table 1

Model	*β*	SE	L_CI	U_CI	*P*	r^{2p}
M1						r^2=0.653
AGE	-0.11	0.020	-0.15	-0.07	**1.09×10^{-8}**	0.025
GENDER	-1.28	0.035	-1.35	-1.21	**1.95×10^{-204}**	0.506
TIME DRAWN	-0.46	0.033	-0.52	-0.39	**3.22×10^{-40}**	0.125
TANNER STAGE	-0.25	0.015	-0.28	-0.22	**1.55×10^{-53}**	0.165
M1 + HEIGHT + WEIGHT						r^2=0.666
AGE	-0.10	0.019	-0.14	-0.07	**6.78×10^{-8}**	0.022
GENDER	-1.25	0.043	-1.33	-1.16	**1.41×10^{-145}**	0.394
TIME DRAWN	-0.45	0.033	-0.52	-0.39	**3.41×10^{-40}**	0.125
TANNER STAGE	-0.23	0.015	-0.26	-0.20	**6.88×10^{-47}**	0.145
HEIGHT	0.10	0.025	0.05	0.15	**4.28×10^{-5}**	0.013
WEIGHT	-0.16	0.024	-0.21	-0.12	**6.95×10^{-12}**	0.035
M2						r^2=0.666
AGE	-0.10	0.019	-0.14	-0.07	**6.61×10^{-8}**	0.022
GENDER	-1.31	0.063	-1.43	-1.18	**7.26×10^{-82}**	0.244
TIME DRAWN	-0.45	0.033	-0.51	-0.39	**5.85×10^{-40}**	0.125
TANNER STAGE	-0.23	0.016	-0.26	-0.20	**2.33×10^{-44}**	0.138
HEIGHT	0.12	0.030	0.06	0.18	**6.43×10^{-5}**	0.012
F-MASS	-0.11	0.020	-0.15	-0.07	**6.32×10^{-8}**	0.012
L-MASS	-0.16	0.039	-0.23	-0.08	**5.77×10^{-5}**	0.022

Table shows regression analyses of different covariates versus CTX in 1325 participants aged 15.5 (580 males, 745 females). M1= adjustment for average age, gender, tanner stage and whether the individual attended the clinic in the morning or afternoon. M1 + HEIGHT + WEIGHT = Model 1 in addition to height and weight; M2 = Model 1 in addition to total-body lean mass and fat mass. n= sample size; SE = standard error; *β* = SD change CTX per SD change in exposure (excluding categorical variables: gender, time drawn and tanner stage); L_CI = lower 95% confidence estimate of *β*; U_CI = upper 95% confidence estimate of *β*; *P* = strength of evidence against the null hypothesis of no association between the outcome and exposure variable; r^{2p} = partial proportion of the variance in the outcome explained by the exposure in the specified model. r^2= total proportion of variance in the CTX explained by the all exposures in the specified model

Supplementary Table 2

							ALSPAC (n=3382)			YFS (n=1558)			GOOD (n=938)		
**LOCUS**	**RSID**	**POS**	**GENE**	**PMID**	**r^2**	**EA**	**β***	**SE**	***P***	**β***	**SE**	***P***	**β***	**SE**	***P***
**8q24.12**	rs4355801	119993054	*TNFRSF11B*	19079262 & 18455228	0.90	A	-0.01	0.021	6.41×10^{-1}	-0.08	0.036	**3.47×10^{-2}**	0.08	0.043	7.17×10^{-2}
	rs7839059	120045723	*TNFRSF11B*	23437003	0.42	A	-0.04	0.022	9.58×10^{-2}	-0.13	0.042	**2.14×10^{-3}**	0.03	0.045	5.58×10^{-1}
	rs2062375	120046973	*TNFRSF11B*	20548944	0.97	C	-0.03	0.021	1.47×10^{-1}	-0.12	0.037	**7.79×10^{-4}**	0.06	0.043	1.48×10^{-1}
	**rs2062377□**	**120076601**	***TNFRSF11B***	**19801982 & 22504420**	**1.00**	**A**	-0.03	0.021	1.75×10^{-1}	-0.10	0.037	**9.00×10^{-3}**	0.06	0.043	1.77×10^{-1}
	rs6469792	120077552	*TNFRSF11B*	19079262	0.74	C	-0.04	0.021	**3.93×10^{-2}**	-0.10	0.036	**3.96×10^{-3}**	0.07	0.043	8.06×10^{-2}
	rs11995824	120081881	*TNFRSF11B*	19801982	0.79	G	-0.04	0.021	**4.53×10^{-2}**	-0.11	0.036	**2.16×10^{-3}**	0.04	0.043	3.07×10^{-1}
	rs6469804	120114010	*TNFRSF11B*	18445777 & 19079262	0.88	A	-0.03	0.021	2.41×10^{-1}	-0.09	0.037	**1.27×10^{-2}**	0.04	0.043	3.15×10^{-1}
	rs6993813	120121419	*TNFRSF11B*	18445777 & 19079262	0.69	C	-0.04	0.021	8.27×10^{-2}	-0.11	0.036	**3.09×10^{-3}**	0.04	0.043	3.89×10^{-1}
**13q14.11**	**rs9533090□**	**41849449**	***AKAP11***	**19801982 & 22504420**	**1.00**	**T**	0.00	0.021	8.23×10^{-1}	0.00	0.036	9.22×10^{-1}	0.03	0.045	5.16×10^{-1}
	rs9594738	41850145	*TNFSF11*	18445777 & 19079262	1.00	T	0.00	0.021	8.29×10^{-1}	0.00	0.036	9.10×10^{-1}	0.03	0.045	5.46×10^{-1}
	rs9533093	41859597	*TNFSF11*	19079262	0.23	T	0.04	0.024	8.60×10^{-2}	0.06	0.046	1.80×10^{-1}	0.06	0.055	2.66×10^{-1}
	rs9594759	41930593	*TNFSF11*	18445777 & 19079262	0.68	T	0.00	0.021	9.71×10^{-1}	-0.01	0.037	7.94×10^{-1}	0.02	0.045	6.06×10^{-1}
	rs1021188□	42014133	*TNFSF11*	21124946 & 23437003	0.00	C	-0.08	0.027	**3.84×10^{-3}**	-0.02	0.056	6.75×10^{-1}	-0.06	0.060	2.93×10^{-1}
**18q21.33**	**rs884205□**	**58205837**	***TNFRSF11A***	**19801982 | 22504420**	**1.00**	**A**	-0.06	0.024	**7.22×10^{-3}**	-0.02	0.041	5.62×10^{-1}	-0.11	0.051	**2.75×10^{-2}**
	rs3018362	58233073	*TNFRSF11A*	19079262	0.68	A	-0.05	0.022	**1.62×10^{-2}**	-0.01	0.037	7.93×10^{-1}	-0.08	0.045	7.18×10^{-2}

Table showing association of previously reported RANK (*TNFRSF11A*), RANKL (*TNFSF11*) and OPG (*TNFRSF11B*) variants with cortical thickness in ALSPAC (n=3382), GOOD (n=938) and Young Finns (n=1558). (POS) = position in the genome based on hg18; (GENE) = closest gene; (PMID) = accession number of the publication in Pubmed which described the association with BMD; (r^2) = the pairwise LD estimate in CEU populations between the SNP in bold and all other SNPs in that locus; (EA) = effect allele; (β*) = effect size; (SE) = standard error of β*; (*P*) = *P*-value; (PMID) = accession number of the publication in Pubmed which described the association with BMD. *Effect estimates expressed as adjusted SD per copy of the effect allele (EA). Note: rs9533090□ is found upstream of *TNFSF11*, but is closest to *AKAP11*. □ - denotes the variants which were used to generate allele scores (i.e. independent signals).

J

Supplementary Table 3

							ALSPAC (n=3382)			YFS (n=1558)			GOOD (n=938)		
**LOCUS**	**RSID**	**POS**	**GENE**	**PMID**	**r^2**	**EA**	**β***	**SE**	***P***	**β***	**SE**	***P***	**β***	**SE**	***P***
**8q24.12**	rs4355801	119993054	*TNFRSF11B*	19079262 & 18455228	0.90	A	-0.05	0.018	**2.74×10^{-3}**	-0.13	0.036	**2.89×10^{-4}**	-0.12	0.044	**5.11×10^{-3}**
	rs7839059	120045723	*TNFRSF11B*	23437003	0.42	A	-0.07	0.019	**1.04×10^{-4}**	-0.14	0.041	**4.88×10^{-4}**	-0.17	0.046	**2.07×10^{-4}**
	rs2062375	120046973	*TNFRSF11B*	20548944	0.97	C	-0.07	0.018	**2.97×10^{-4}**	-0.16	0.036	**1.16×10^{-5}**	-0.14	0.044	**2.21×10^{-3}**
	**rs2062377□**	**120076601**	***TNFRSF11B***	**19801982 & 22504420**	**1.00**	**A**	-0.06	0.018	**1.27×10^{-3}**	-0.16	0.037	**2.17×10^{-5}**	-0.11	0.044	**1.14×10^{-2}**
	rs6469792	120077552	*TNFRSF11B*	19079262	0.74	C	-0.05	0.018	**5.92×10^{-3}**	-0.14	0.036	**1.45×10^{-4}**	-0.10	0.044	**1.81×10^{-2}**
	rs11995824	120081881	*TNFRSF11B*	19801982	0.79	G	-0.05	0.018	**2.66×10^{-3}**	-0.14	0.036	**7.79×10^{-5}**	-0.14	0.045	**1.39×10^{-3}**
	rs6469804	120114010	*TNFRSF11B*	18445777 & 19079262	0.88	A	-0.05	0.018	**3.25×10^{-3}**	-0.15	0.037	**2.68×10^{-5}**	-0.12	0.044	**8.67×10^{-3}**
	rs6993813	120121419	*TNFRSF11B*	18445777 & 19079262	0.69	C	-0.05	0.018	**5.38×10^{-3}**	-0.15	0.036	**5.40×10^{-5}**	-0.14	0.044	**1.30×10^{-3}**
**13q14.11**	**rs9533090□**	**41849449**	***AKAP11***	**19801982 & 22504420**	**1.00**	**T**	-0.02	0.018	2.09×10^{-1}	-0.11	0.035	**1.48×10^{-3}**	0.01	0.046	8.98×10^{-1}
	rs9594738	41850145	*TNFSF11*	18445777 & 19079262	1.00	T	-0.02	0.018	2.09×10^{-1}	-0.11	0.035	**1.47×10^{-3}**	-0.01	0.046	9.01×10^{-1}
	rs9533093	41859597	*TNFSF11*	19079262	0.23	T	-0.01	0.021	7.59×10^{-1}	-0.04	0.046	3.56×10^{-1}	-0.01	0.055	8.66×10^{-1}
	rs9594759	41930593	*TNFSF11*	18445777 & 19079262	0.68	T	-0.01	0.018	6.20×10^{-1}	0.01	0.037	8.67×10^{-1}	0.04	0.046	3.79×10^{-1}
	rs1021188□	42014133	*TNFSF11*	21124946 & 23437003	0.00	C	-0.12	0.024	**1.97×10^{-7}**	-0.16	0.056	**3.10×10^{-3}**	-0.30	0.063	**1.65×10^{-6}**
**18q21.33**	**rs884205□**	**58205837**	***TNFRSF11A***	**19801982 & 22504420**	**1.00**	**A**	-0.09	0.021	**3.05×10^{-5}**	0.02	0.040	5.95×10^{-1}	-0.04	0.051	4.90×10^{-1}
	rs3018362	58233073	*TNFRSF11A*	19079262	0.68	A	-0.07	0.019	**1.93×10^{-4}**	-0.02	0.036	5.35×10^{-1}	-0.06	0.046	2.07×10^{-1}

Table showing association of previously reported RANK (*TNFRSF11A*), RANKL (*TNFSF11*) and OPG (*TNFRSF11B*) variants with cortical BMD in ALSPAC (n=3382), GOOD (n=938) and Young Finns (n=1558). (POS) = position in the genome based on hg18; (GENE) = closest gene; (PMID) = accession number of the publication in Pubmed which described the association with BMD; (r^2) = the pairwise LD estimate in CEU populations between the SNP in bold and all other SNPs in that locus; (EA) = effect allele; (β*) = effect size; (SE) = standard error of β*; (*P*) = *P*-value; (PMID) = accession number of the publication in Pubmed which described the association with BMD. *Effect estimates expressed as adjusted SD per copy of the effect allele (EA). Note: rs9533090□ is found upstream of *TNFSF11*, but is closest to *AKAP11*. □ - denotes the variants which were used to generate allele scores (i.e. independent signals).

Supplementary Table 4

							ALSPAC (n=3382)			YFS (n=1558)			GOOD (n=938)		
LOCUS	RSID	POS	GENE	PMID	r^2	EA	β*	SE	P	β*	SE	P	β*	SE	P
**8q24.12**	rs4355801	119993054	*TNFRSF11B*	19079262 & 18455228	0.90	A	0.03	0.016	6.18x10^{-2}	0.12	0.036	**1.08x10^{-3}**	0.02	0.035	6.70x10^{-1}
	rs7839059	120045723	*TNFRSF11B*	23437003	0.42	A	0.02	0.017	1.94x10^{-1}	0.05	0.041	2.44x10^{-1}	0.03	0.037	3.58x10^{-1}
	rs2062375	120046973	*TNFRSF11B*	20548944	0.97	C	0.03	0.016	6.34x10^{-2}	0.08	0.036	**1.96x10^{-2}**	-0.01	0.035	8.37x10^{-1}
	**rs2062377□**	**120076601**	***TNFRSF11B***	**19801982 & 22504420**	**1.00**	**A**	0.02	0.016	2.74x10^{-1}	0.09	0.037	**1.46x10^{-2}**	-0.01	0.035	8.34x10^{-1}
	rs6469792	120077552	*TNFRSF11B*	19079262	0.74	C	0.01	0.016	5.88x10^{-1}	0.08	0.036	**1.90x10^{-2}**	0.01	0.035	8.47x10^{-1}
	rs11995824	120081881	*TNFRSF11B*	19801982	0.79	G	0.01	0.016	5.34x10^{-1}	0.08	0.036	**2.22x10^{-2}**	0.01	0.036	6.77x10^{-1}
	rs6469804	120114010	*TNFRSF11B*	18445777 & 19079262	0.88	A	0.02	0.016	3.03x10^{-1}	0.09	0.037	**9.92x10^{-3}**	0.00	0.035	9.38x10^{-1}
	rs6993813	120121419	*TNFRSF11B*	18445777 & 19079262	0.69	C	0.01	0.016	5.01x10^{-1}	0.09	0.036	**1.20x10^{-2}**	0.02	0.035	6.58x10^{-1}
**13q14.11**	**rs9533090□**	**41849449**	***AKAP11***	**19801982 & 22504420**	**1.00**	**T**	-0.02	0.016	3.36x10^{-1}	0.08	0.036	**1.71x10^{-2}**	0.00	0.037	9.42x10^{-1}
	rs9594738	41850145	*TNFSF11*	18445777 & 19079262	1.00	T	-0.02	0.016	3.37x10^{-1}	0.08	0.035	**1.95x10^{-2}**	0.01	0.037	8.46x10^{-1}
	rs9533093	41859597	*TNFSF11*	19079262	0.23	T	-0.02	0.018	2.60x10^{-1}	0.07	0.046	1.31x10^{-1}	-0.01	0.044	8.67x10^{-1}
	rs9594759	41930593	*TNFSF11*	18445777 & 19079262	0.68	T	-0.01	0.016	5.20x10^{-1}	0.03	0.036	3.75x10^{-1}	-0.04	0.037	3.06x10^{-1}
	rs1021188□	42014133	*TNFSF11*	21124946 & 23437003	0.00	C	0.05	0.020	**9.21x10^{-3}**	0.04	0.056	5.27x10^{-1}	0.06	0.050	2.08x10^{-1}
**18q21.33**	**rs884205□**	**58205837**	***TNFRSF11A***	**19801982 & 22504420**	**1.00**	**A**	0.05	0.018	**7.03x10^{-3}**	0.04	0.040	3.26x10^{-1}	0.02	0.041	6.53x10^{-1}
	rs3018362	58233073	*TNFRSF11A*	19079262	0.68	A	0.04	0.017	**2.91x10^{-2}**	0.02	0.036	6.15x10^{-1}	0.05	0.037	1.83x10^{-1}

Table showing association of previously reported RANK (*TNFRSF11A*), RANKL (*TNFSF11*) and OPG (*TNFRSF11B*) variants with Periosteal Circumference in ALSPAC (n=3382), GOOD (n=938) and Young Finns (n=1558). (POS) = position in the genome based on hg18; (GENE) = closest gene; (PMID) = accession number of the publication in Pubmed which described the association with BMD; (r^2) = the pairwise LD estimate in CEU populations between the SNP in bold and all other SNPs in that locus; (EA) = effect allele; (β*) = effect size; (SE) = standard error of β*; (*P*) = *P*-value; (PMID) = accession number of the publication in Pubmed which described the association with BMD. *Effect estimates expressed as adjusted SD per copy of the effect allele (EA). Note: rs9533090□ is found upstream of *TNFSF11*, but is closest to *AKAP11*. □ - denotes the variants which were used to generate allele scores (i.e. independent signals).
